# Association of skewed X-chromosome inactivation with FMR1 CGG repeat length and anti-Mullerian hormone levels: a cohort study

**DOI:** 10.1186/s12958-017-0250-9

**Published:** 2017-04-28

**Authors:** David H. Barad, Sarah Darmon, Andrea Weghofer, Gary J. Latham, Filipovic-Sadic, Qi Wang, Vitaly A. Kushnir, David F. Albertini, Norbert Gleicher

**Affiliations:** 1 The Center for Human Reproduction (CHR), New York, NY USA; 2 The Foundation for Reproductive Medicine, New York, NY USA; 3 Department of Obstetrics and Gynecology, Vienna University School of Medicine, Vienna, Austria; 4 Asuragen, Austin, TX USA; 5 Department of Obstetrics and Gynecology, Wake Forest University, Winston Salem, NC USA; 6 Department of Molecular and Integrative Physiology, University of Kansas Medical Center, Kansas, USA; 7 Stem Cell and Molecular Embryology Laboratory, The Rockefeller University, New York, NY USA

**Keywords:** FMR1, Primary Ovarian Insufficiency, Skewed X-chromosome inactivation, AMH, Methylation, Ovarian reserve, POF

## Abstract

**Background:**

Premutation range CGGn repeats of the FMR1 gene denote risk toward primary ovarian insufficiency (POI), also called premature ovarian failure (POF). This prospective cohort study was undertaken to determine if X-chromosome inactivation skew (sXCI) is associated with variations in FMR1 CGG repeat length and, if so, is also associated with age adjusted antimüllerian hormone (AMH) levels as an indicator of functional ovarian reserve (FOR).

**Methods:**

DNA samples of 58 women were analyzed for methylation status and confirmation of CGG_n_ repeat length. Based on previously described FMR1 genotypes, there were 18 women with *norm FMR1* (both alleles in range of CGG_*n*=26–34_), and 40 women who had at least one allele at CGG_n<26_ or CGG_>34 *(*_ *not-norm FMR1*). As part of a routine evaluation of ovarian reserve, patients at our fertility center have their serum AMH assessed at first visit. Regression models were used to test the association of ovarian reserve, as indicated by serum AMH, with sXCI.

**Results:**

sXCI was significantly lower among infertility patients with *norm FMR1* (6.5 ± 11.1, median and IQR) compared to those with *not-norm FMR1* (12.0 ± 14.6, *P* = 0.005), though among young oocyte donors the opposite effect was observed. Women age >30 to 38 years old demonstrated greater ovarian reserve in the presence of lower sXCI as evidenced by significantly higher AMH levels (GLM sXCI_10%, f = 11.27; *P* = 0.004).

**Conclusions:**

Together these findings suggest that FMR1 CGG repeat length may have a role in determining X-chromosome inactivation which could represent a possible mechanism for previously observed association of low age adjusted ovarian reserve with FMR1 variations in repeat length. Further, larger, investigations will be required to test this hypothesis.

## Background

Clinically the *FMR1* gene (Xq27.3) is currently primarily associated with the Fragile X syndrome (FXS), characterized by expansion of CGG_n_ in the 5′UTR region to CGG_n>200_. FXS is considered the most common cause of familial mental retardation and autism. Risk screening for FXS is based on *FMR1* mutations defined by a normal or common range (CGG_n<45_), an intermediate or gray zone range between approximately CGG_*n*=45–54_ and a premutation range of approximately CGG _*n*=55–200_. The later can expand within one generation to full mutation length [1].

Tassone et al. reported that full mutation *FMR1* carriers demonstrate decreased fragile X mental retardation protein (FMRP) and increased *FMR1* mRNA [2]. The premutation range phenotype has been hypothesized to be the consequence of toxicity of accumulating FMRP protein or *FMR1* mRNA transcripts [3–5], and is clinically characterized by significantly increased risk toward POI [6],, with a reported prevalence of 16 to 24% among women in the premutation range [7].

Why only a minority of premutation carriers develop POI is unknown. Moreover, over the last decade it has become apparent that more subtle forms of POI, so called occult POI, also appears to be associated with certain CGG_n_ ranges, leading to the conclusion that the *FMR1* gene in some fashion is associated with speed of follicle loss in ovaries [7]. Others have suggested that, after adjustment for CGG_n_, race, smoking, body mass index, and method of ascertainment, additional genes in combination with *FMR1* may be responsible for emergence of the POI phenotype [8].

Fu et al. described the distribution of CGG_n_ in the normal population to peak around CGG_*n*=29–30_ [9]. Based on this observation, we hypothesized that this very large population peak represented a potentially normal CGG_n_ range of the *FMR1* gene’s ovarian function and, indeed, described a normal (*norm*) range of CGG_*n*=26–34_, which allowed for the definition of abnormally *low*_CGGn<26_, and *high*_CGGn>34_ mutations. If both X alleles are in normal range they are considered *norm*; if one allele is in and the other outside normal range they are considered heterozygous (*het*), and homozygous (*hom*) if both alleles are outside normal range. *Het* and *hom* mutations were further subdivided based on whether abnormal alleles were *high* or *low* [10–12].

In a series of cross sectional studies [10–13] and a longitudinal study [14], we were able to describe associations between these newly defined *FMR1* mutations and ovarian aging patterns, leading to the hypothesis that the *FMR1* gene affects functional ovarian reserve (FOR) at different ages and, therefore, affects ovarian aging.

The previously noted long known association between premutation range CGG_n_ and POI in humans [6] also supports an ovarian function of the *FMR1* gene, as does a recently reported mouse homologue, which offers further evidence that the gene is involved in ovarian aging [15]. A recently published cross-sectional study of considerable size was, however, unable to find associations between age of natural menopause and number of CGG repeats in traditional normal and intermediate ranges [16].

Though how the *FMR1* gene affects ovarian aging remains unknown, we have hypothesized that different mutations in the gene may affect recruitment speed of primordial (or resting) follicles [17]. In drosophila *fmr1* related microRNAs have been associated with primordial germ-line cell suppression and have been described as extrinsic factors for germ-line stem cell maintenance [18]. FMRP has been noted to form a complex with PIWI, a maternal component of the polar granule, a germ-plasm-specific organelle essential for drosophila germline specification [19].

Interestingly, among human *FMR1* premutation carriers, POI is dependent upon mutation length, though the relationship is not linear since maximum risk of POI appears to occur among women in CGG_*n*=80–100_ range. This is approximately the mid-point of the premutation range of CGG_n~55–200_.

Since epigenetic modifications figure prominently in the development of *FMR1* syndromes, structural changes in CGG_n_ as well as epigenetic effects via methylation and histone modifications can result in transcriptional silencing [20].

X-chromosome inactivation (XCI) in the female achieves dosage compensation with males, and leads to differences in epigenetic markings on the active and inactive X-chromosome [21]. Due to XCI, all females are mosaics with random inactivation of either the maternally or paternally derived X-chromosome [22]. In normal females, 50% of the CpG promoter sites of genes subject to XCI are methylated, though they are unmethylated in normal males. Approximately 15% of genes on the inactive X-chromosome escape inactivation [23]. When XCI is not random, there is an imbalance of cells expressing either the paternal or maternal X-chromosome, known as sXCI [24]. Inactivation of the entire X-chromosome involves many additional specialized factors, histone variants and chromatin modifiers [25].

The present study was undertaken to determine how variations in CGGn repeat length may relate to sXCI, and whether sXCI of the *FMR1* gene may be associated with changes in FOR, as assessed by AMH levels.

## Methods

We prospectively assessed 70 reproductive age women, 55 infertility patients (age 36.9 ± 5.5 years) presenting to our Center for IVF treatment, and 15 young oocyte donors (age 24.5 ± 2.4), with a high performance *FMR1* PCR and with serum AMH levels. We purposely overrecruited patients with CGG repeats outside our defined normal range of CGG_*n*=26–34._ Identical alleles are more common within this normal range and in those individuals sXCI could not be assessed. Censoring those individuals left 58 subjects for the first part of this analysis. We further restricted the study group in a second analysis evaluating ovarian reserve to those subjects without evidence of AMH above 5 (75th percentile in young women) [26]. This left 50 subjects for that portion of the analysis.

### CGG sizing and methylation PCR

DNA samples were analyzed for methylation status and confirmation of CGG_n_ repeat length using AmplideX® *FMR1* mPCR Reagents (Asuragen, Austin, TX) per the manufacturer’s recommended protocol. Briefly, DNA samples were separately aliquoted to a control or methylation-sensitive digestion reaction. Products of the control digestion reaction were amplified using FAM-labeled primers, whereas products of the methylation-sensitive reaction were amplified using HEX-labeled primers. The percent methylation for each allele was calculated as the proportion of signal in the HEX- and FAM channels, normalized to reference control signals. The mPCR assay determines both CGG_n_ and the methylation status of each allele [27].

Methylation leads to XCI, and is expected to be randomly (50:50) distributed between each X chromosome. Results using the mPCR assay were normally distributed (P > 0.05, Shapiro-Wilk test with Benjamin-Hochberg correction) [28], and repeated measurements demonstrated low variance (Table 1) with at least 95% of the average of all possible pairwise combinations of technical replicates falling within 5% of the mean methylation value for *FMR1* alleles that best represented random XCI. Thus, all study samples were run in replicate using mPCR, and the mean values were used for statistical analyses. In this analysis, the X chromosome with the lower CGG_n_ allele is defined as “X1” and the one with the higher CGG_n_ as allele “*X*2”. The extent to which the actual observed distribution deviates from 50:50 is measured as the mean skew of X-chromosome inactivation (sXCI). sXCI is, thus, calculated as [ABS (50 - observed percent methylation of X1) + ABS (50 - observed percent methylation of *X*2)]/2. When both alleles were identical, the sXCI could not be calculated since we could not identify methylation of the individual FMR1 alleles. sXCI was not normally distributed. We compared quantitative values of sXCI with the Mann-Whitney *U* test and created a categorical variable for sXCI with cut-off at 10% skew, the minimum skew that can be supported within the known technical variance of the mPCR assay.

**Table 1:** Variance of mPCR measurements over a range of 1–86% methylation

CGG#	Average % Methylation (*n* = 14)	Std Dev
18	3%	1%
30	59%	4%
32	5%	1%
56	38%	4%
85	1%	1%
116	86%	11%
>200	2%	1%

Values near 50% methylation (38–59%) demonstrated standard deviations of 4%

*FMR1* alleles representing 7 distinct expansion lengths were assessed in 14 independent mPCR runs using multiple operators to establish the technical variation of the assay

A primary goal of this analysis was to compare the methylation patterns relative to previously defined *FMR1* genotypes [12]. We, thus, set out to recruit women from our existing patient pool with *norm FMR1* (CGG_*n*=26–34_) and with at least one CGG_n<26_ or CGG_n>34,_ (*not-norm*). Proportions of *norm* and not-*norm* study subjects, therefore, are not expected to reflect previously reported percentages in normal populations [29]. Women with known sex chromosome aneuploidy were excluded from the study.

### AMH

The second goal of this study was to examine the possible association between sXCI and FOR, using AMH as an indicator of FOR. We excluded from this part of the analysis participants with known causes of extreme changes in FOR, such as polycystic ovary syndrome, or with known low FOR (LFOR), such as ovarian dysgenesis, previous oophorectomy, chemotherapy or advanced ovarian age.

As part of a routine evaluation of ovarian reserve, patients at our fertility center have their serum AMH assessed at first visit. AMH was assayed by a single commercial laboratory (Esoterix, Calabasas Hills, California) using an enzymatically amplified two-site immunoassay AMH Gen II ELISA ref A73818, (Beckman Coulter Brea, CA). For women, whose AMH levels were undetectable, the AMH level was set to 0.15 ng/mL the lowest detectable level with this assay system.

Since AMH is right skewed, to approach normality, we utilized the natural logarithm of AMH in applied regression models. Since AMH is known to vary with age [26, 30], all models were also adjusted for age.

Regression models tested included a general linear model (GLM), adjusted for age, in which we created a categorical variable sXCI_10% for less than or equal to 10% skew or greater than 10% skew, the minimum skew that can be supported within the known technical variance of the mPCR assay, (Table 1), to determine how AMH varied in presence of greater or lesser sXCI. To test for interaction with age we created a categorical variable grouping subjects in three age groups with cut-off at 30 and at 38 years old. We focused on the 30 to 38 year old group since this is the time in a woman’s reproductive life of rapid change in ovarian reserve and we were interested in the effect of sXCI during that transitional time.

The GLM models tested were:

Ln (AMH) = β0 + β1 sXCI_10% + β2 Age group + β3 sXCI_10% × Age group + random error

Ln (AMH) = β0 + β1 sXCI_10% + β2 Age + random error

We also ran a Mann-Whitney *U* test within the three age groups to confirm these findings.

### Data

The datasets during and/or analyzed during the current study available from the corresponding author on reasonable request.

### Statistics

Normality was tested by Kolmogorov-Smirnov test. Quantitative variables were presented as mean ± standard deviation (SD) or geometric mean and 95% confidence intervals, and qualitative variables as number (%). Normally distributed variables were compared by GLM ANOVA. The Mann-Whitney *U* test was used to analyze non-normal quantitative variables and results presented as median (IQR = interquartile range). All statistical analyses were carried out with the use of the Statistical Package for the Social Sciences 21.0 (IBM SPSS). *P* < 0.05 was considered statistically significant.

## Results

A total of 70 women were initially recruited for this study. Excluding those with identical *FMR1* alleles left 58 subjects; 46 infertility patients, 12 with *norm FMR1* (both alleles at CGG_*n*=26–34_) and 34 with at least one allele at CGG_n<26_ or CGG_>34_ (*not-norm FMR1*), *and* 12 donors, 6 *norm* and 6 *not-norm.*

The data were further restricted in the analysis of effects of sXCI on ovarian reserve. Four infertility patients and 4 egg donors with AMH greater than 5 ng/mL [31], who were thought to have polycystic ovaries, were also censored which left 50 women of reproductive age as study population for the second portion of the analysis.

Table 2 summarizes the characteristics among the infertility patients and young egg donors in this analysis. As expected, donors were younger and had higher AMH compared to infertility patients. Not surprisingly, donors recruited into this analysis had a higher percentage of normal CGG_n_ genotypes. However, this dataset was based on intended over recruiting of women outside CGG_*n*=26–34_, and does not reflect the natural distribution of CGG repeats in the general population. sXCI was noted to be significantly higher among donors with CGG_*n*=26–34_ compared to infertility patients with *norm* (CGG_*n*=26–34_) (*P* = 0.022), though there was no significant difference of sXCI between the infertility patients and donors in the *not-norm* (CGG_n<26_ or CGG_n> 34_) category.

**Table 2:** Characteristics among the infertility patients and young egg donors in this analysis

	Infertility Patients (*n* = 46)	Donors (*n* = 12)	*P*
Age (years)	36.5 ± 5.2	24.7 ± 2.4	<0.001
AMH ng/mL^a^	0.3 (2.0)	4.4 (5.1)	<0.001
CGG_X1^a^	29 (7)	29 (7)	0.745
CGG2_X2^a^	31.5 (6)	31.5 (4)	0.484
CGG			
Norm	12 (26.1%)	6 ((50.0%)	-
Any Low	18 (39.1%)	4 (33.3%)	-
Any High	14 (30.4%)	2 (16.7%)	-
Low/High	2 (4.3%)	0 (0%)	-
Methylation%_X1	48.7 ± 17.4	50.7 ± 19.4	0.740
Methylation%_X2	50.5 ± 20.6	49.8 ± 19.3	0.834
Median sXCI^a^ (All)	10.0 (11.0)	11.5 (11.5)	0.824
*Norm* CGG_26 - 34_^a^	6.5 (11.1) (*n* = 12)	19.8 (7.9) (*n* = 6)	0.003
*Not-Norm* CGG _<26_or CGG_>34_^a^	12.0 (14.6) (*n* = 34)	9.3 (9.4) (*n* = 6)	0.127

^a^Median (IQR); Mann Whitney *U* test

### sXCI and CGG_n_

**Fig. 1:**
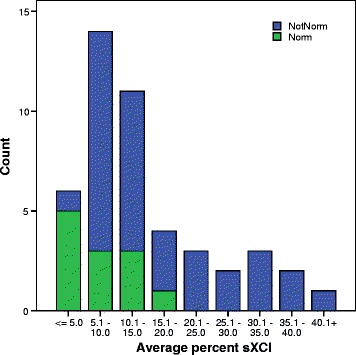
Distribution of sXCI among 46 infertility patients categorized by FMR1 repeat status. sXCI was significantly lower among 12 infertility patients with *norm* (CGG_*n*=26–34_) _compared_ to 34 others with *not-norm CGG* (at least one CGG_n<26_ or CGG_n>34_) (Z = −2.80, *P* = 0.005)

Results for sXCI were significantly different between infertility patients and egg donors. Using the Mann-Whitney *U* test sXCI was significantly lower among 12 infertility patients with *norm* (CGG_*n*=26–34_) compared to 34 others with *not-norm CGG* (at least one CGG_n<26_ or CGG_n>34_) (Z = −2.80, *P* = 0.005) (Fig. 1). While among egg donors the sXCI was higher among 6 *norm* women compared to 6 CGG *not-norm* women (z = −2.69, *P* = 0.026), though not significantly so when adjusted for multiple comparison.

### Ovarian reserve

Median AMH in the whole study population was 0.935 ng/mL, with range from undetectable (<0.15 ng/mL) to 13 ng/mL. AMH decreased with age of women, consistent with widely reported findings in the literature [26, 30]. Median AMH among the 50 women in the AMH restricted study population was 0.35 ng/mL, with range from undetectable (<0.15 ng/mL) to 4.5 ng/mL.

### SXCI and ovarian reserve

**Fig. 2:**
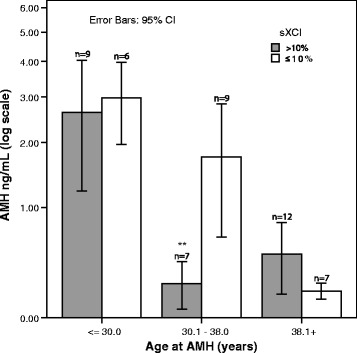
Bar graph of AMH against Age categorized by sXCI of 10%. Bar graph of geometric mean and 95% confidence intervals of AMH (natural log scale) in three age strata with cut-off at age 30 and 38 years comparing participants in each stratum with ≤ 10% sXCI to those with >10% sXCI. In a GLM analysis stratified by age-groups there was a significant interaction between sXCI and age group (*p* = 0.004). While there is no significant difference in AMH observed for women < 30 or for women > 38 years old there is a significant difference in the AMH levels for those age >30 to 38 (*n* = 16). In this age group those with ≤ 10% sXCI (*n* = 7) demonstrated a significantly higher AMH level compared to those with > 10% sXCI (*n* = 9) (GLM sXCI_10%, f = 11.27; *P* = 0.004)

sXCI was compared in relationship to AMH in 50 women (42 infertility patients and 8 oocyte donors) who demonstrated AMH levels ≤ 5.0 ng/mL. In a GLM analysis of the effect of Age and sXCI on lnAMH we noted a significant interaction between Age and sXCI (*p* = 0.004). Accordingly we analyzed the three age subgroups individually and found that there was a highly significant effect of sXCI on mean AMH among the 16 women who were > 30 to 38 years old: sXCI > 10%, AMH 0.2, 95% CI 0.118 to 0.340 and sXCI ≤ 10%, AMH 1.74, 95% CI 0.67 to 2.83 (F11.53, *p* = 0.004), while there was no observable effect of sXCI among the 15 women ≤ 30 years old (F = 1.06, *p* = 0.32) or the 19 women > 38 years old (F = 3.35, *p* = 0.085) (Fig. 2). The Mann-Whitney *U* test yielded similar findings.

## Discussion

In this study, we report the association of CGG_n_ with sXCI, and association of variations in observed sXCI with variations in serum AMH, an important marker of FOR. Combined, the findings, therefore, provide a possible mechanism to explain previously observed associations of the *FMR1* gene with variations in FOR.

Among women with *FMR1* premutation range CGG_n_, alterations in mRNA production have been suggested as a possible cause of premature ovarian failure, while no such abnormalities of mRNA production have been described for women with what is widely considered the common (i.e., normal) range of CGG_n<55_.

In the present study, we found that, as CGG_n_ deviates from CGG_*n*=26–34_ up or down, sXCI will increase. The observation that CGG_n_ on both sides of CGG_*n*=30_ appears related to methylation skew offers an interesting new possibility of how CGG_n_, within the currently considered common (normal) range, may affect *FMR1* function. These findings also support our prior clinical reports in which we found increased likelihood of LFOR in infertile women with increasing distance in both directions from CGG_*n*=29–31_ [10, 14, 17, 32].

The major effector of X inactivation is an RNA gene known as Xist (X-inactive specific transcript) [21]. Untranslated RNA transcribed from the Xist gene coats the inactive X chromosome, leading to its silencing [33, 34]. X inactivation occurs very early in embryo development, around the time of implantation [35].

Since evidence presented here suggests that CGG_n_ of the *FMR1* gene appears associated with sXCI, how the *FMR1* gene’s CGG_n_ might affect initiation of Xist inactivation of one X chromosome raises further interesting questions. Interaction with Xist has previously also been reported for the *BRCA1* gene,^31,32^ in itself an interesting finding, as we [36] and others [37] reported distinct CGG_n_ distribution patterns in the *FMR1* gene in *BRCA* mutation carriers.

Skewing in favor of larger proportions of normally active X chromosomes has been previously noted in women with full mutations [27, 38] and premutation carrier females [39]. In contrast we are here describing such a pattern in the *FMR1* gene with fewer than 55 CGG repeats (CGG_n<55_).

In this analysis, the overall median skew was 12%. Most investigators consider the threshold for highly skewed X-chromosome inactivation to lie at 80 to 90% [40]. Others have reported that among 220 unaffected normal females the mean distribution of X-chromosome inactivation was 50:50. Only 9.5% were considered highly skewed with a threshold of 90, and 23.6% with a threshold of 80% [41].

These numbers raise question about the clinical significance of our observed variations in sXCI since these differences cannot be considered highly skewed.

Observations may, however, be age dependent: In leucocytes of adult females, the full fragile X mutation was found more often on the inactive X chromosome, but less so in younger females [42]. We observed an opposite relationship between sXCI and CGG_n_ among young oocyte donors compared to older infertility patients. Our analysis noted differences between young oocyte donors and older infertile patients in the significantly higher percentage of sXCI in women with *FMR1* alleles outside normal range (i.e., CGG_n<26_ or CGG_n>34_) than in *norm* range (CGG_*n*=26–34_). Among older infertility patients, we observed increased sXCI in women with CGG_n_ outside *norm* range, while younger donors did not demonstrate sXCI differences between *norm* and *not-norm* women. The importance of CGG_n_ for sXCI may, therefore, increase with advancing female age.

We find further evidence of age dependence in that the effects of sXCI were only significant within the >30 to 38-year age group of women (Fig. 2). This is, of course, the age in which ovarian reserve first begins to decline and when genetic and environmental effects on ovarian reserve may first become apparent. Among younger women there is sufficient redundancy of functional ovarian reserve to mask these effects while among older women there is generally universal decline. Thus, it is during the transitional time in the 30’s when subtle effects on ovarian reserve may be most apparent.

One limitation of this analysis is that this is a relatively small study group mostly comprised of infertility patients with prior evidence of low functional ovarian reserve. Our findings in a small group of healthy egg donors were quite different from those observed in the infertility patients. Thus, these findings may not be generalizable to all women.

Our observation, that a higher sXCI is associated with lower AMH levels, offers a potential insight concerning how deviations from *norm* CGG_*n*=26–34_ may affect FOR.

For technical reasons, such conclusions have, however, to be viewed with a degree of caution: *FMR1* genotyping was performed in this study from peripheral blood. Tissue-specific differences in CGG_n_ have been reported in fragile X affected men and women [43], and mosaicism of CGG_n_ and methylation is well established [44]. Observations made in peripheral blood, at least theoretically, may, therefore, not reflect the genetic and epigenetic make-up of ovaries.

One can also hypothesize that skewed X inactivation might influence early gametogenesis, leading later in life to differences in FOR. However others have found that in women with premutation range CGG_n_, POI was not associated with increased skewing [41, 45]. Within the traditionally normal range of CGG_n < 55_, sXCI may, however, indeed be associated with such changes.

## Conclusions

In summary, we report the association of increased low-level sXCI of the *FMR1* gene with deviations from *norm* CGG_n_ (CGG_*n*=26–34)_. This association was primarily observed in older infertility patients but not in younger oocyte donors. Among infertility patients of mid-reproductive age, we observed that sXCI greater than 10% was associated with lower levels of age-adjusted AMH. Together, these observations support previously reported effects of *FMR1* genotypes and sub-genotypes on FOR [32, 46], warranting further explorations of the *FMR1* gene in reference to FOR at various ages.

## Abbreviations

AMH: Antimüllerian hormone
CGGn: Number of cytosine guanine guanine repeats
FMR1: Fragile X Mental Retardation 1 gene
FMRP: Fragile X mental retardation protein
FOR: Functional ovarian reserve
FXS: Fragile X syndrome
PCR: Polymerase chain reaction
POF: Premature ovarian failure
POI: Premature ovarian insufficiency
sXCI: X-chromosome inactivation skew
XCI: X-chromosome inactivation
